# Effectiveness of a transactional model-based education programme for enhancing stress-coping skills in industrial workers: a randomized controlled trial

**DOI:** 10.1038/s41598-023-32230-2

**Published:** 2023-03-28

**Authors:** Mohammad Hossein Kaveh, Farnaz Mehrazin, Rosanna Cousins, Hamidreza Mokarami

**Affiliations:** 1 Department of Health Education and Promotion, School of Health, Shiraz University of Medical Sciences, Shiraz, Iran; 2 Research Center for Health Science, Institute of Health, Shiraz University of Medical Sciences, Shiraz, Iran; 3 Department of Health Education and Promotion, School of Health, Shiraz University of Medical Sciences, Shiraz, Iran; 4 Department of Psychology, Liverpool Hope University, Liverpool, UK; 5 Department of Ergonomics, School of Health, Shiraz University of Medical Sciences, PO Box 71645-111, Shiraz, Iran

**Keywords:** Human behaviour, Health occupations

## Abstract

The aim of this study was to examine the effectiveness of a stress management educational intervention programme based on the Transactional Model of Stress and Coping (TMSC) among industrial workers. Participants were 106 employees of a power plant in Iran, randomly assigned into an intervention group and a control group. The intervention comprised active and participatory methods to enhance employees coping skills and it was delivered in six face-to-face sessions. Data was collected using the Ways of Coping Questionnaire, the Multidimensional Scale of Perceived Social Support, the Perceived Stress Scale, and the Spiritual Well-Being Scale at baseline and three months after the intervention. We found mean scores of distancing, self-controlling, seeking social support, escape-avoidance, planned problem-solving, positive reappraisal, total coping skills, perceived social support, and spiritual well-being significantly differed at follow-up compared to baseline in the intervention group, but not in the control group. There was also a significant difference in the mean score of perceived stress between the two groups. We conclude that the educational intervention based on the TMSC was effective in improving coping skills and reducing perceived stress. We suggest that interventions based on the TMSC model can be supportive in workplaces where job stress is common.

## Introduction

Employees are exposed to a range of physical and psychosocial risks at their workplaces according to occupation^1^. Job stress is associated with various negative outcomes at both individual and organizational levels. There is consensus in the literature that occupational stress can be described as a perceived imbalance of the demands of a job and the individual’s resources to meet those requirements^2,3^. Similarly, adequate management of a potential gap between the one’s capabilities and the demands of the job is an ability to cope^3^, and failing to cope with one’s work demands has the potential for harmful physical and emotional outcomes as a result of chronic triggering of the stress response^2^. There is a significant accumulation of evidence of the physiological processes that show how stress mediates the onset and impact of various disease states, and similarly, there is evidence of a connection of psychosocial stressors supporting negative health outcomes, and dysfunction in the workplace^4^. These problems, in turn, increase the rates of absenteeism and presenteeism, poor work performance, accident and injury, and low productivity^5^.

Stress management is a poorly defined concept^6^ and thus a variety of practical techniques have been used to cope with occupational and life stressors to prevent or ameliorate negative physical and psychological consequences^7^. In practice, stress management interventions can be used at the organisational level, at the individual level, and the interface of individual and organisation^8^. Similarly, distinctions have been made regarding primary, secondary and tertiary interventions^7^, with a strong argument that a risk assessment approach to manage stressors in the workplace is a legal duty for employers, and thus has to be a part of the stress management actions^4^. Nevertheless, although organisational changes can be made to ameliorate stressors in some workplaces^9^, there are also inherent stressors in some occupations, and there are differences in how individuals perceive events as stressful^3,10^. That is, subjective factors are critical to the experience of stress. Factors such as self-efficacy, optimism, social support from co-workers, supervisors and family, and participation in decision-making have positive effects on coping with stressors in the workplace^11^. Critically, there are substantial benefits for an organisation through empowering employees with skills to cope with inherent stressors from both work and life. Whilst it is not appropriate to establish training programmes that simply serve to adapt workers to poorly designed job^2^, equally, stress management methods should not be abandoned when they seek to tackle secondary, individual aspects of coping with stressors on a job in a dynamic workplace^7,12^. Following from this, we discerned a need to develop an effective education programme for industrial workers through reference to established scientific research in the area of stress and coping. There is a strong business case for promoting mental health in the workplace, and providing coping skills training can be considered a practical and cost-effective method to reduce job stress and increase the psychological well-being of employees, ultimately increasing their physical and mental health^5^.

The conceptualisation of coping in the context of stress has for many years revolved around the work of Lazarus and Folkman. Their Transactional Model of Stress and Coping (TMSC) illustrates how stress and coping is a process based on changing cognitive appraisals of a given situation and one’s resources^3^. Appraisals (primary and secondary) and coping efforts are the two main components of a dynamic transactional process between the person and their environment. If their situation is perceived as threatening, then the person appraises their ability to deal with that threat, and from there takes a coping strategy that is largely problem-focused or emotion-focused. Essentially then, coping has two major functions: to regulate harmful emotions, and to change the negative person-environment relationship that has produced the distress^13^. The appropriateness and quality of coping efforts determines outcomes, including adaptation, emotional wellness, functional status, or behaviour change, accepting that there are several variables can mediate the process of coping. In particular, social support and meaning-based thinking, like positive reappraisal and spiritual beliefs, are among the important mediating factors that should be considered when planning education programmes to support the development of stress coping skills [3. 10. 12].

There is evidence to suggest that educational health promotion programmes are effective interventions for stress management in the workplace. For example, a rational-emotive health education intervention used to coach technical college teachers’ in Nigeria, significantly decreased the stress of the intervention group compared to a control group^14^. Similarly, using a sample of distressed managers across several work sectors, Asplund et al.^15^ showed that a guided internet-based stress management intervention in a randomized controlled trial reduced stress and improved other mental-related and work-related health symptoms compared to a control group both post-test and at a six-month follow-up. Also, an evaluation of a mobile-app based stress management intervention found a reduction in perceived stress in highly stressed workers, if no improvement in anxiety, depression or work engagement^16^. Relatively few studies, however, have used the TMSC as a framework for managing stress in the workplace. One exception is a quasi-experimental study of healthcare workers in Iran (n = 84) in which half the sample received a transactional model-based education programme^17^. When compared to controls, follow-up scores of the intervention group indicated significantly improved coping strategies. A second study examined the efficacy of an education programme delivered on the internet to a sample of 26 American teachers to manage burnout^18^. One week after the 4-week education programme was completed, there was a significant increase in healthy coping strategies as measured by the Maslach Burnout Inventory-Educator Survey for the intervention group and no change in the control group (n = 25). The authors suggest that their study confirms that the TMSC framework provides an appropriate approach to develop interventions to increase healthy coping strategies in the teaching profession.

To our knowledge, there remains a need to provide supportive stress interventions beyond the teaching profession, including employees working in large industrial power plants. To our knowledge, no study has assessed the effect of a transactional model-based education programme on stress management and coping skills of industrial workers. Therefore, the purpose of this study was to investigate the effect of a health promotion educational intervention based on the TMSC on stress management and coping skills among industrial workers in Iran.

## Method

The Scientific and Ethics Committee of Shiraz University of Medical Sciences approved the study protocol (IR.SUMS.REC.1399.372). The research was conducted in accordance with the Declaration of Helsinki, and all participants provided written informed consent.

### Study design and participants

This study used a randomized controlled trial (RCT) design with a 1:1 ratio. Participants were employees of a combined cycle power plant in Iran. The company had 211 employees at the time of the study, all employees worked one of two teams, each working rotating 12-h shifts. Inclusion criteria were having more than one year of job tenure, no history of stress management education, and no history of psychiatric treatment, determined through participants’ self-report. Exclusion criteria included absence from two educational sessions, not attending the pre-test or post-test sessions, withdrawal from the study, and undergoing treatment by a psychiatrist due to mental illness during the intervention. 194 employees were eligible to participate, however some declined to participate leaving a consenting sample of 170 employees. A sample size calculation was calculated based on the effect size (0.58) provided by Faryabi et al.^17^ which indicated that a 1:1 group size of 48 was sufficient at an alpha set at 0.05, and a beta of 0.2 / power of 0.80. To account for potential atrophy at follow-up, we increased recruitment by 10%, and recruited 53 to the two groups in the study. To prevent or mitigate contamination and bias in this per-protocol study, we randomly selected one work shift as the intervention group and the other as the control group using a tossed coin. Random allocation of potential participants from each shift was then undertaken using an Excel spreadsheet programme that permitted sampling by shuffling the population^19^. Hence a random sample of 53 employees was allocated to the study from the consenting sample in each of the two shifts.

**Figure 1:**
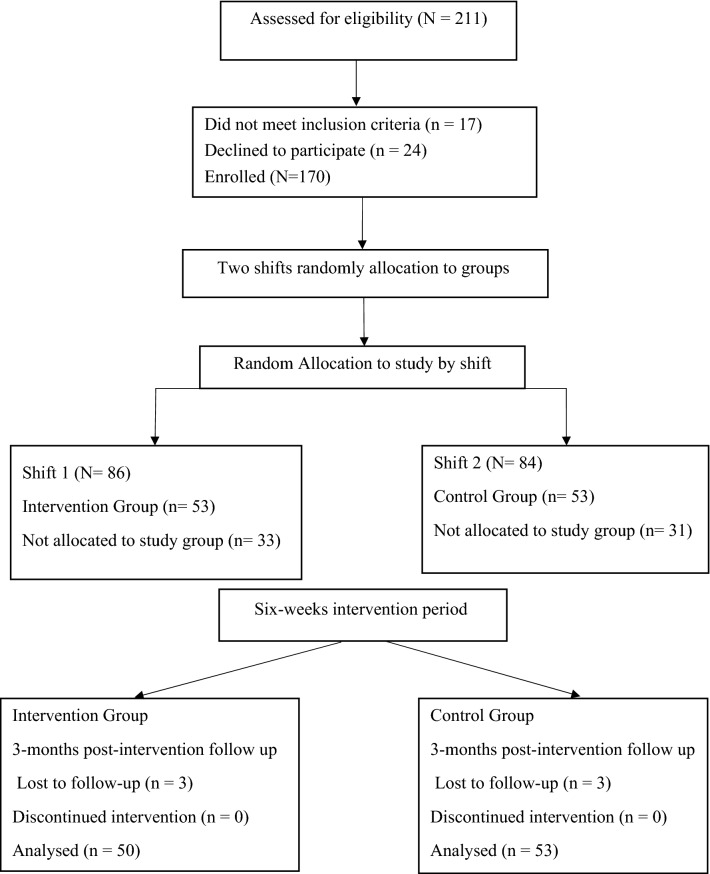
Consort flow diagram of the study. 211 employees who participants worked one of two 12-hour shifts in an industrial plant were invited into the study. 170 gave informed consented. One shift was randomly allocated as the educational intervention group the other was the control group. From potential participants 53 participants were randomly allocated into each study group. Three participants dropped out of the Intervention Group before the three-months post-intervention follow-up which was on the same day for both groups. Thus, data was analysed from 50 participants in the Intervention group and compared with 53 participants in the control group.

Data was collected at baseline, and three months after the 6-weeks educational intervention period had been completed. All participants completed the anonymous study questionnaires on the same day at baseline, and the same day at the follow-up, except for three participants in the intervention group who did not provide data at the three-month follow-up. This gave a final sample for data analysis of 103 participants. Figure 1 shows the flow of the participants throughout the study, based on the Consolidated Standards of Reporting Trials (CONSORT)^20^.

## Measures

### Demographic information

Demographic variables—age, sex, marital status, education level, job tenure, and employment status—were assessed at baseline.

### Ways of coping questionnaire (WCQ)

WCQ is an established measure based on Lazarus and Folkman’s process-oriented stress and coping theory. It was originally developed with 68 items to identify the coping strategies people use in stressful situations^2^. Further work from this lab identified eight types of emotion- and problem-based coping, across 66 items. These are confrontive coping, distancing, self-controlling, seeking social support, accepting responsibility, escape-avoidance, planned problem-solving, and positive reappraisal^13^. Various versions of the questionnaire exist according to the setting in which it is being used. In this study participants were first asked about a stressful situation related to work that occurred during the previous week. Participants were then asked how extensively they used a given coping strategy in relation to that situation using a four-point scale from 0 (not used) to 3 (used a great deal) for each of the 66 items. Responses are totalled for each of the eight subscales and the total WCQ. The psychometric properties of the Persian version of this questionnaire have been confirmed^22^. Cronbach’s alpha coefficient was 0.89 for the total questionnaire in the study.

### Multidimensional scale of perceived social support (MSPSS)

MSPSS^23^ is a brief questionnaire of perceived adequacy of social support. It includes 12 items that enquire after the perceived adequacy of family, friends and significant others (four items for each subscale), using a seven-point Likert scale (1 = very strongly disagree, 7 = very strongly agree). A higher score equates with higher perceived social support. The psychometric properties of the Persian version of this questionnaire have been confirmed^24^. Cronbach’s alpha coefficient for the questionnaire was 0.82.

### Perceived stress scale (PSS-10)

PSS-10^25^ is a tool that measures the extent to which life events are appraised as stress provoking in terms of overload, lack of control and poor predictability. It is widely used in many settings including general population and occupational investigations. Each of the ten items asks how often in the past month participants have been stressed by a situation. For example, upset about an unexpected event, or confident about their ability to hand a personal problem. All items were scored using a five-point Likert scale (0 = never, 4 = very often) and the four positively worded items were reverse scored, such that higher scores indicate higher stress. The psychometric properties of the Persian version of PSS-10 were confirmed^26^. In this study Cronbach’s alpha coefficient was 0.91.

### Spiritual well-being scale (SWBS)

SWBS^27^ is a 20-item indicator of the quality of life in the sphere of spiritual well-being. It includes 10 items associated with religious well-being, and one’s relationship with God, and 10 items measuring existential well-being, and a one’s understanding of the purpose of life. The SWBS uses a six-point Likert-type scale (1 = strongly agree, 6 = strongly disagree), with some items reversed so that high scores indicate more well-being. The SWBS provides three scores; in this study only the total SWBS score was used. The psychometric properties of the Persian version of the scale were confirmed^28^. Cronbach’s alpha coefficient was 0.78 for the total questionnaire.

## Educational intervention programme

The intervention consisted of six face-to-face educational sessions held for the intervention group—which was divided into six subgroups. Each of the six weekly sessions lasted 1.5–2 h. The training programme is outlined in Table 1. A WhatsApp group was created for each subgroup to provide supplementary information and short video clips.

**Table 1:** Content of educational session.

Session	Session title (content)	Teaching–learning method(s)	Instructional aids	Target constructs in TMSC
1	Definition of stress and job stress, the destructive effects of stress on physical and mental health, and performance sensitization to the importance and controllability of stress	Lecture and question/answer	Whiteboard and powerpoint	Perceived susceptibility and severity
2	The importance of motivational relevance and motivational congruence	Lecture and question/answer	Powerpoint	Primary appraisal (motivational relevance and motivational congruence)
3	Explaining the causes and factors aggravating stress, effects of personality traits on stress, and the role of social support in stress coping	Group discussion and lecture	Powerpoint	Social support
4	Describing emotion-focused coping and problem-focused coping styles, differences between these coping styles, and how to use coping strategies in different situations	Lecture, group discussion, question/ answer, and role play	Powerpoint, pamphlets, posters, and educational clips	Secondary appraisal (emotion-focused coping and problem-focused coping)
5	Feedback on the previous session’s assignment, description of positive reappraisal coping, and spiritual beliefs	Lecture, group discussion, question/answer, and role play	Powerpoint, pamphlet, whiteboard, and motivational clip	Positive reappraisal and spiritual beliefs
6	Overviewing the previous sessions and group discussion	Lecture, group discussion, and question/answer	Powerpoint	Total structures of model

All the educational content was provided to the control group after the analysis of the intervention.

## Data analysis

In both evaluation phases, histograms and the Kolmogorov–Smirnov test were first used to check the distribution and skewness of data. The dependent variables were all normally distributed, thus, chi-square, t-tests, and analyses of covariance (ANCOVA) were used to compare the data. All analyses were performed using SPSS version 23 software (SPSS Inc., Chicago, IL, USA). A *p*-value ≤ 0.05 represented statistical significance.

## Results

Mean age was 35.88 (SD = 5.22) years and ranged from 27 to 50 years in the intervention group (n = 50); mean age was 36.42 (SD = 5.99) years and ranged 25 to 52 years in the control group (n = 53). Mean job tenure ranged from 1 to 27 years with an average of 8.34 (SD = 6.47) years in the intervention group and ranged from 1 to 28 years with an average of 8.92 (SD = 6.24) years in the control group. Table 2 shows the baseline demographic characteristics of the study participants. The distributions of age, marital status, education level, job tenure, and employment status did not significantly differ between the two groups at baseline. As there was a difference between the two groups regarding sex, this variable was included in subsequent analyses as a covariate.

**Table 2:** Demographic characteristics of intervention and control groups.

Variable	Intervention group (n = 50)	Control groups (n = 53)	*p* value
	n (%)	n (%)	
Age (year)			
≥ 35	25 (50)	22 (41.5)	0.387a
> 35	25 (50)	31 (58.5)	
Job tenure (year)			
5 ≥	27 (54)	22 (41.5)	0.205a
> 6	23 (46)	31 (58.5)	
Sex			
Women	8 (16)	1 (1.9)	0.014b
Men	42 (84)	52 (98.1)	
Education level			
Associate	6 (12)	16 (30.2)	0.076a
Bachelor	31 (62)	25 (47.2)	
MSc and above	13 (26)	12 (22.6)	
Marital status			
Married	38 (76)	42 (79.2)	0.693a
Single	12 (24)	11 (20.8)	
Employment status			
Permanent	26 (52)	22 (41.5)	0.286a
Temporary	24 (48)	31 (59.5)	

^a^Pearson Chi-Square; ^b^Fisher's Exact Test.

Statistical analyses showed that mean scores of confrontive coping (t = − 0.226, *p* = 0.822), distancing (t = 0.438, *p* = 0.662), self-controlling (t = − 0.345, *p* = 0.730), seeking social support (t = − 0.187, *p* = 0.852), accepting responsibility (t = − 1.60, *p* = 0.113), escape-avoidance (t = 0.151, *p* = 0.881), planned problem-solving (t = − 0.313, *p* = 0.755), positive reappraisal (t = 0.510, *p* = 0.611), total WCQ score (t = − 0.324, *p* = 0.747), perceived social support (t = − 0.450, *p* = 0.654), and spiritual well-being (t = 0.985, *p* = 0.327) did not significantly differ between two groups at baseline (see Table 3). However, at the three-month follow-up, a significant change in terms of coping strategies, spiritual well-being, and perceived social support was observed in the intervention group compared to the control group. Statistical analyses showed that the mean scores of distancing (t = 6.01, *p* < 0.001), self-controlling (t = 2.84, *p* = 0.005), seeking social support (t = 5.70, *p* < 0.001), escape-avoidance (t = − 2.17, *p* = 0.032), planned problem-solving (t = 5.12, *p* < 0.001), positive reappraisal (t = 6.07, *p* < 0.001), total WCQ score (t = 5.17, *p* < 0.001), perceived social support (t = 2.77, *p* = 0.007), and spiritual well-being (t = 4.06, *p* < 0.001) were significantly different between the two groups at the three-month follow-up.

**Table 3:** Comparison of mean scores of variables at baseline and at 3‐month follow‐up according to group.

Variables	Time	Intervention M ± SD	Control M ± SD	*p* value (t-test)	*p* value (Ancova)
Confrontive coping	Baseline	6.76 ± 2.45	6.86 ± 2.39	0.822	0.761a
	Follow-up	7.46 ± 2.14	6.94 ± 2.52	0.267	0.234b
	*p* value (Paired t-test)	0.135	0.869		
Distancing	Baseline	6.8 ± 2.31	6.60 ± 2.23	0.662	0.589a
	Follow-up	9.68 ± 2.65	6.54 ± 2.63	< 0.001	< 0.001b
	*p* value (Paired t-test)	< 0.001	0.877		
Self-controlling	Baseline	9.58 ± 3.14	9.79 ± 3.09	0.730	0.728a
	Follow-up	11.86 ± 2.70	10.01 ± 3.75	0.005	0.004b
	*p* value (Paired t-test)	< 0.001	0.694		
Accepting responsibility	Baseline	5.88 ± 1.95	6.50 ± 2.03	0.113	0.144a
	Follow-up	6.44 ± 1.56	6.56 ± 2.34	0.751	0.851b
	*p* value (Paired t-test)	0.073	0.878		
Seeking social support	Baseline	9.54 ± 4.20	9.67 ± 3.31	0.852	0.973a
	Follow-up	14.84 ± 4.01	10.37 ± 3.93	< 0.001	< 0.001b
	*p*-value (Paired t-test)	< 0.001	0.219		
Escape-Avoidance	Baseline	7.12 ± 3.39	7.01 ± 3.41	0.881	0.947a
	Follow-up	4.84 ± 3.74	6.39 ± 3.52	0.032	0.026b
	*p* value (Paired t-test)	< 0.001	0.26		
Planned problem solving	Baseline	9.00 ± 3.34	9.18 ± 2.75	0.755	0.927a
	Follow-up	12.32 ± 2.86	9.20 ± 3.27	< 0.001	< 0.001b
	*p* value (Paired t-test)	< 0.001	0.969		
Positive reappraisal	Baseline	11.08 ± 4.29	10.69 ± 3.26	0.611	0.507a
	Follow-up	16.50 ± 3.55	11.69 ± 4.40	< 0.001	< 0.001b
	*p* value (Paired t-test)	< 0.001	0.083		
Total WCQ	Baseline	86.56 ± 21.79	87.84 ± 18.56	0.747	0.909a
	Follow-up	111.68 ± 19.21	89.49 ± 23.91	< 0.001	< 0.001b
	*p* value (Paired t-test)	< 0.001	0.629		
Perceived stress	Baseline	19.48 ± 4.32	21.21 ± 4.32	0.020	0.016a
	Follow-up	14.36 ± 5.33	19.4 ± 4.32	< 0.001	< 0.001b
	*p* value (Paired t-test)	< 0.001	0.008		
Perceived social support	Baseline	61.20 ± 12.33	62.32 ± 12.92	0.654	0.858a
	Follow-up	71.66 ± 12.52	63.32 ± 17.50	< 0.001	0.007b
	*p* value (Paired t-test)	< 0.001	0.702		
Spiritual well-being	Baseline	108.94 ± 19.26	105.22 ± 19.00	0.327	0.325a
	Follow-up	125.42 ± 17.14	110.07 ± 22.89	< 0.001	< 0.001b
	*p* value (Paired t-test)	< 0.001	0.110		

**a**: Adjusted for Sex by ANCOVA; **b**: Adjusted for Sex and baseline value by ANCOVA.

Paired-samples t-tests revealed that, unlike the control group, the mean score difference (MSD) of distancing (MSD = 2.88, t = − 6.60, *p* < 0.001), self-controlling (MSD = 2.28, t = − 4.04, *p* < 0.001), seeking social support (MSD = 5.30, t = − 7.10, *p* < 0.001), escape-avoidance (MSD = 2.28, t = 3.70, *p* < 0.001), planned problem-solving (MSD = 3.32, t = 6.48, *p* < 0.001), positive reappraisal (MSD = 5.42, t = − 7.13, *p* < 0.001), total WCQ (MSD = 25.12, t = − 6.43, *p* < 0.001), perceived social support (MSD = 10.46, t = − 5.57, *p* < 0.001), and spiritual well-being (MSD = 16.48, t = − 6.85, *p* < 0.001) at the three-month follow-up were significantly different from the mean scores at baseline in the intervention group. In both intervention and control groups the mean score of perceived stress was significantly reduced at the three-month follow-up compared to baseline. However, the MSD was 1.87 in the control group and 4.92 in the intervention group, showing a statistically significant difference (t = − 2.96, *p* = 0.004).

Finally, we performed ANCOVA to be sure that sex and baseline values did not significantly change the results. As can be seen in Table 3, there was no significant change in the results.

## Discussion

This randomized controlled trial assessed the effect of an educational intervention based on the TMSC on stress management and coping skills among industrial workers in Iran. The results suggest that the educational intervention could significantly improve coping skills strategies, spiritual well-being, and perceived social support, and decrease perceived stress among industrial workers. This original study is the first to use an educational intervention based on the robust TMSC to help industrial workers cope with stress through the development of appropriate stress coping skills.

The significant impact of the educational programme on coping skills in the present study aligns with previous research in other working populations^17,18^. A review of extant literature confirmed that no study has particularly addressed educational interventions based on the TMSC to promote coping skills in industrial workers. Among the studied coping skills, the biggest improvement in the intervention group was observed in positive reappraisal. This emotion-regulation strategy essentially involves reinterpreting an experience to deliberately seek out positive meaning when a situation has previously been recognised as negative for self^29^. A positive reappraisal is often the first step towards re-engagement with a stressor event, and developing such coping strategies enables a person to reduce the negative impact of stressors in their life^30^.

Seeking social support was another coping skill that was increased in the intervention group. This problem-focused coping skill intentionally pursues practical, informational and emotional support from relevant others to relieve the particular stressor which is challenging in one’s life^31^. Positive perceptions of one’s social support is associated with better psychological well-being, physical health, and quality of life, and have been shown to buffer individuals against the adverse effects of stressful events^31^. In this regard, the results indicated a significant improvement in perceived social support in the intervention group. The educational programme designed to improve seeking social support, small group discussions, and the WhatsApp social media group may have helped improve group dynamism and promote perceived social support among the workers in the intervention group.

The present study showed a remarkable change in spiritual well-being when the measure was repeated after the intervention. Drawing upon one’s beliefs and understanding of the holistic purpose of life can be recognised as adaptive, and a positive coping strategy in the face of considerable suffering^32^. This underlying factor is not considered in the original TMSC model. Nevertheless, in the present study, the effect of the educational intervention on spiritual well-being was evaluated using a valid and standard tool, and was seen to make an important contribution to stress coping skills. This is in line with arguments that spiritual well-being is important, and a predictor of the levels of subsequent happiness, psychological well-being, and stress. That is, spiritual well-being has a significant impact on stress coping skills and health promotion behaviours^32–34^.

One of the most important results of the present study was that the educational programme reduced perceived stress even when measured three months after the intervention, suggesting some longevity to the principles of the TMSC programme. We acknowledge that an assertion that this programme could empower industrial workers to cope with stressors in the long term requires additional data collection, perhaps after two years, as it is well-known that there are skills decay over time, even when used regularly^35^. It has been suggested that a regular ‘refresher’ educational session may provide sustainability for the long-term for skills programmes^36^. Critically, there is evidence that this is so from a skills-based training programme for midwives which found that a two-session refresher course provided one-year after the initial training minimised the decay four months after the refresher course, compared with participants who did not have the refresher course^37^. Altogether, additional follow-up in future studies will further endorse the benefits of providing education programmes for enhancing stress-coping skills in industrial workers, and whether refreshers are additionally supportive.

The present study is the first to describe the effect of an educational intervention based on the comprehensive and standard method of TMSC among industrial workers. This RCT study produced causal evidence of the role stress coping skills training can contribute to industrial workers’ well-being. In this study, we measured spiritual well-being in addition to eight coping skills of the TMSC model. The simultaneous use of face-to-face and virtual education is one of the other strengths of the study.

Despite the strengths of the study, it also has some limitations. This study was conducted in one Iranian industrial company and may not generalize to other industrial work environments, or culturally diverse environments. Self-reported questionnaires were used to gather study variables, which may have been accompanied by some bias despite the full explanation provided to the participants at the beginning of the study as an attempt to gain their trust. The lack of long-term outcome assessment may also be considered another limitation of the present study. Finally, despite trying to prevent or mitigate contamination, selecting one work shift as the intervention group and the other as the control group might be contamination.

## Conclusion

This study provides evidence of the effectiveness of an educational intervention based on TMSC for improving coping skills and spiritual well-being. The improvement in coping skills had a positive effect on increasing perceived social support and reducing perceived stress. Altogether, these results suggest that an educational intervention programme based on a systematic framework, such as the TMSC, and on-site interventions can effectively improve employees' quality of life. Therefore, it is suggested that educational interventions be implemented based on standard and valid models such as TMSC in other work environments given the ubiquity of job stress.

## Data Availability

Data associated with this study is available upon reasonable request to the corresponding author.
